# 

**Published:** 1894-01

**Authors:** 


### CONTENTS.

### *EDITORIALS.*

| | PAGE |
|---|---|
| Appeal for Payment, . . . . . . . . . . . . . | 1 |
| Isopathy, . . . . . . . . . . . . . . . | 2 |

### *MISCELLANEOUS.*

| | |
|---|---|
| Staphylorraphy. Edmund Carleton, M. D., . . . . . . . | 4 |
| A Case of Dermoid Cyst of the Parotid Gland, with Specimen. Edmund Carleton, M. D., . . . . . . . . . . | 6 |
| Tuberculinum. Horace P. Holmes, M. D., . . . . . . . | 8 |
| Ringworm, . . . . . . . . . . . . . . . | 15 |
| Provings and Clinical Observations with High Potencies. Malcolm Macfarlan, M. D., . . . . . . . . . . . | 16 |
| A Hint or Two. C. Carleton Smith, M. D., . . . . . . | 24 |
| Erotomania During Typhoid Fever. C. M. Boger, M. D., . . . | 25 |
| In Memoriam—Samuel Swan, M. D. Thomas Skinner, M. D., . . | 26 |
| Address to Delinquents in the December Number. One of the Delinquents, . . . . . . . . . . . . . | 27 |
| The Conditions of Nux Vomica in Whitlow. E. W. Berridge, M. D., | 28 |
| In Memoriam—John C. Robert, M. D., . . . . . . . | 28 |
| How to Cure Rhus Poisoning. A Layman, . . . . . . | 29 |

### *BOOK NOTICES.*

| | |
|---|---|
| Outlines of Practical Hygiene, . . . . . . . . . | 29 |
| The Physician's Visiting List, . . . . . . . . . | 30 |
| Funk & Wagnalls' Standard Dictionary of the English Language, . | 30 |
| International Medical Annual for 1894, . . . . . . . | 31 |
| American Text-Book of Gynecology, . . . . . . . . | 32 |
| Diseases of Women, . . . . . . . . . . . . | 32 |

### *NOTES AND NOTICES.*

| | |
|---|---|
| Southern Pines, Moore County, North Carolina—Professional Repartee —Musical Contest, . . . . . . . . . . | 32 |

